# Gene Expression in Pancreatic Cancer-Like Cells and Induced Pancreatic Stem Cells Generated by Transient Overexpression of Reprogramming Factors

**DOI:** 10.3390/jcm10030454

**Published:** 2021-01-25

**Authors:** Chika Miyagi-Shiohira, Issei Saitoh, Masami Watanabe, Hirofumi Noguchi

**Affiliations:** 1 Department of Regenerative Medicine, Graduate School of Medicine, University of the Ryukyus, Okinawa 903-0215, Japan; chika@med.u-ryukyu.ac.jp; 2 Division of Pediatric Dentistry, Graduate School of Medical and Dental Science, Niigata University, Niigata 951-8514, Japan; isaito@dent.niigata-u.ac.jp; 3 Department of Urology, Okayama University Graduate School of Medicine, Dentistry and Pharmaceutical Sciences, Okayama 700-8558, Japan; masami5@md.okayama-u.ac.jp

**Keywords:** induced fibroblast-like (iF) cells, induced pluripotent stem (iPS) cells, induced tissue-specific stem (iTS) cells, reprogramming factors, pancreatic cancer, epithelial-mesenchymal transition (EMT)

## Abstract

We previously reported that transient overexpression of reprogramming factors can be used to generate induced pluripotent stem (iPS) cells, induced tissue-specific stem (iTS) cells, and fibroblast-like (iF) cells from pancreatic tissue. iF cells have tumorigenic ability and behave similarly to pancreatic cancer cells. In this study, we analyzed gene expression in iF cells and iTS-P cells (iTS cells from pancreatic tissue) via microarray analysis and quantitative reverse transcription-polymerase chain reaction (qRT-PCR). The expression levels of the Mybl2 and Lyn genes, which are reported to be oncogenes, were significantly higher in iF cells than in iTS-P cells. The expression level of Nestin, which is expressed in not only pancreatic progenitor cells but also pancreatic ductal adenocarcinomas, was also higher in iF cells than in iTS-P cells. Itgb6 and Fgf13, which are involved in the pathogenesis of diseases such as cancer, exhibited higher expression levels in iF cells than in iTS-P cells. Unexpectedly, the expression levels of genes related to epithelial-mesenchymal transition (EMT), except Bmp4, were lower in iF cells than in iTS-P cells. These data suggest that the Mybl2, Lyn, Nestin, Itgb6, and Fgf13 genes could be important biomarkers to distinguish iTS-P cells from iF cells.

## 1. Introduction

Pluripotent stem (PS) cells hold great promise for the development of stem cell therapies to treat diverse human diseases [1,2,3,4,5,6,7,8,9,10,11,12]. However, numerous limitations hinder the clinical application of embryonic stem (ES) cells and induced pluripotent stem (iPS) cells, including the tumorigenic risk of transplanted undifferentiated cells [13,14,15,16,17]. Recently, we focused on developing a method for generating induced tissue-specific stem (iTS) cells by transient transfection of reprogramming factors and subsequent tissue-specific selection [18,19]. Our group generated mouse iTS cells from the pancreas (iTS-P cells) [18,19,20,21] and liver (iTS-L cells) [18], human iTS cells from mesenchymal cells [22] and human-induced tissue-specific progenitor cells from the pancreas (iTP cells) [23]. Other groups recently generated induced tissue-specific stem/progenitor cells through the transient expression of YAP/TAZ [24] and induced endodermal stem/progenitor cells using defined small molecules [25]. Notably, iTS cells were unable to generate teratomas when transplanted subcutaneously into immunodeficient mice. These cells expressed tissue-specific markers and differentiated into each tissue more frequently than ES cells upon differentiation induction. It has been shown that, after reprogramming of mouse/human iPS cells, epigenetic memory is inherited from the parental cells [26,27,28,29,30,31]. On the other hand, we generated induced fibroblast-like (iF) cells simultaneously with iTS-P cells. iF cells showed a morphology similar to that of fibroblasts, unlike iTS-P cells, and had the ability to form tumors, a behavior similar to that of pancreatic cancer cells [32]. By overexpressing reprogramming factors, somatic cells acquire the properties of self-renewal along with unlimited proliferation and exhibit global alterations in the transcriptional program, which are also critical events during carcinogenesis [33].

It has been reported that the development and progression of pancreatic cancer is caused by gene mutations, dedifferentiation, epigenetic changes, activation of oncogenes, and/or epithelial-mesenchymal transmission (EMT) [34,35] and that transient expression of reprogramming factors in vivo results in the development of tumors consisting of undifferentiated dysplastic cells [36]. In this study, we compared gene expression in iTS-P cells and iF cells via microarray analysis and quantitative reverse transcription-polymerase chain reaction (qRT-PCR) to confirm which genes determine the differences between iTS-P cells and iF cells.

## 2. Materials and Methods

### 2.1. Mice and Cell Culture

All of the mouse studies were approved by the review committee of the Okayama University Graduate School of Medicine, Dentistry and Pharmaceutical Sciences, and the Graduate School of Medicine, University of the Ryukyus. Twenty-four-week-old C57/BL6 mice (CLEA Japan, Inc., Tokyo, Japan) were used for primary pancreatic tissue preparation. Each mouse pancreas was digested with 2 mL of cold M199 medium containing 2 mg/mL collagenase (Roche Boehringer Mannheim, Basel, Switzerland). The digested tissues were cultured in Dulbecco’s modified Eagle’s medium (DMEM; Thermo Fisher Scientific, Inc. Tokyo, Japan) supplemented with 10% to 20% fetal bovine serum (FBS; BIOWEST, Riverside, MO, USA). Eight-week-old nonobese diabetic/severe combined immunodeficiency (NOD/SCID) mice (Charles River Laboratories Japan, Inc., Kanagawa, Japan) were used for the teratoma formation studies. iTS-P and iF cells were maintained in complete ES cell medium supplemented with 15% FBS (Millipore, Darmstadt, Germany) on feeder layers of mitomycin C-treated STO cells, as described previously [20]. iTS-P and iF cells were passaged every 5 days.

### 2.2. Plasmid Construction and Transfection

To generate the OSKM plasmid, complementary DNA (cDNA) encoding POU domain, class 5, transcription factor 1 (Pou5f1 or Oct3/4), sex determining region Y-box 2 (Sox2), Kruppel-like factor 4 (Klf4), and the c-myc proto-oncogene (c-Myc) were linked (in that order) with the 2A peptide and inserted into a plasmid containing the cytomegalovirus (CMV) promoter. The OSKM plasmid was transfected on days 1, 3, 5, and 7 into pancreatic cells from 24-wk-old mice, as previously described [8]. The colonies were manually picked 30 to 45 days after the first transfection. The detailed protocol has been described previously [18].

### 2.3. Teratoma/Tumor Formation

A total of 1 × 10^6^ iF cells (passage 20) were inoculated into one thigh of each NOD/SCID mouse. The same number of iTS-P cells (passage 20) was transplanted into the other thigh of each NOD/SCID mouse.

### 2.4. Hematoxylin–Eosin Staining

After fixation with 4% paraformaldehyde, specimens were embedded in paraffin, and 3- to 5-mm-thick sections were stained with Mayer’s hematoxylin and eosin.

### 2.5. Microarray Analysis

Total RNA from iF cells (passage 30), ES cells, iTS-P cells (passage 30), and pancreatic tissue cells (>95% islets) was labeled with biotin. Samples were hybridized with the GeneChip 3′IVT PLUS Reagent Kit (Affymetrix, Tokyo, Japan) and GeneChip Hybridization, Wash and Stain Kit (Affymetrix) according to the manufacturer’s protocol. Arrays were scanned with the GeneChip Scanner 3000 7G system (Affymetrix). Data were analyzed using the Affymetrix GeneChip Command Console software program (Affymetrix).

### 2.6. qRT-PCR

Total RNA was extracted from cells using an RNeasy Mini Kit (Qiagen, Tokyo, Japan). After quantification by spectrophotometry, 2.5 mg of RNA was heated at 85 °C for 3 min and was then reverse-transcribed into cDNA in 25 mL of solution containing 200 units of Superscript II Ribonuclease H-Reverse Transcriptase (Ribonuclease H-RT) (Thermo Fisher Scientific, Inc.), 50 ng of random hexamers (Thermo Fisher Scientific, Inc.), 160 mmol/L dNTPs, and 10 nmol/L dithiothreitol. The thermal cycling parameters were as follows: 10 min at 25 °C, 60 min at 42 °C, and 10 min at 95 °C. Quantification of messenger RNA (mRNA) levels was performed using a TaqMan real-time PCR system according to the manufacturer’s instructions (Applied Biosystems, Foster City, CA, USA). PCR was performed for 40 cycles and included initial steps of 2 min at 50 °C and 10 min at 95 °C. In each cycle, denaturation was performed for 15 s at 95 °C, and annealing/extension was performed for 1 min at 60 °C. PCR was carried out in 20 mL of solution using cDNAs synthesized from 1.11 ng of total RNA. For each sample, the mRNA expression level was normalized by dividing it by the Gapdh expression level. The mouse primers are commercially available (Assays-on-Demand Gene Expression Products; Applied Biosystems). (Sox2: Mm03053810_s1, Oct3/4: Mm03053917_g1, Mycn: Mm00476449_m1, Reg3b: Mm00440616_g1, Reg1: Mm00485651_m1, Reg3g: Mm00441127_m1, Mafa: Mm00845206_s1, Sox9: Mm00448840_m1, Mybl2: Mm00485340_m1, Lyn: Mm01217488_m1, Akt3: Mm00442194_m1, Tnfrsf22: Mm00445826_m1, Nes: Mm00450205_m1, Grem1: Mm00488615_s1, Ctnnd2: Mm00435342_m1, Itgb6: Mm01269869_m1, Fgf13: Mm00438910_m1, Twist1: Mm00442036_m1, Twist2: Mm00492147_m1, Sparcl1: Mm00447784_m1, Efemp1: Mm00524588_m1, Bmp4: Mm00432087_m1, Tgfbr1: Mm00436964_m1, Tgfbr3: Mm00803538_m1, Tgfbi: Mm01337605_m1, Mest: Mm00485003_m1, Dlk1: Mm00494477_m1, Epcam: Mm00493214_m1, Wnt5a: Mm00437347_m1, Gapdh: Mm99999915-g1). The results were obtained from independent experiments performed in triplicate.

### 2.7. Statistical Analyses

The data are presented as the mean ± standard error values. Repeated measures analysis of variance was used for intergroup comparisons. *p* values of <0.05 were considered to indicate statistical significance.

## 3. Results

### 3.1. Generation of iPS, iTS-P and iF Cells from Mouse Pancreatic Tissue

**Figure 1:**
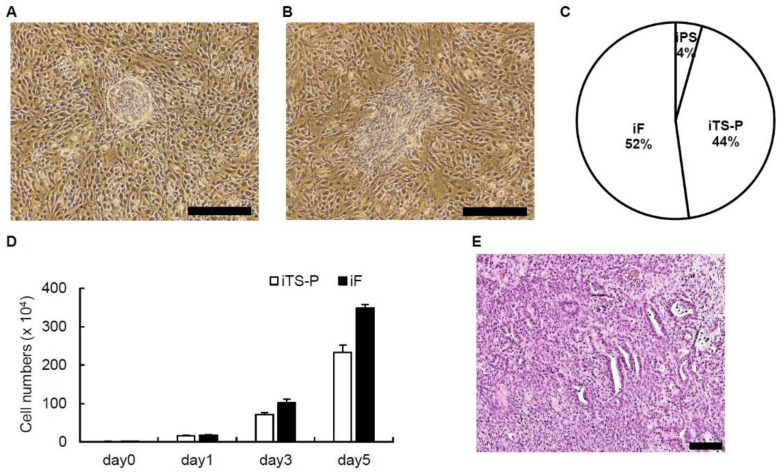
Generation of mouse induced tissue-specific stem (iTS) cells and induced fibroblast-like (iF) cells. (**A**) The morphology of mouse iTS-P cells. Scale bars = 500 µm. (**B**) The morphology of mouse iF cells. Scale bars = 500 µm. (**C**) Percentages of iPS, iTS-P, and iF cells forming colonies. The OSKM plasmid vector was transfected into mouse pancreatic tissue, and colonies were counted after 30–45 days. *n* = 5. (**D**) Proliferation of iTS-P cells and iF cells. (**E**) Hematoxylin and eosin staining of tumors derived from iF cells. Scale bar = 100 µm.

We transfected a single plasmid expressing Oct3/4, Sox2, Klf4 and c-Myc into pancreatic tissue obtained from 24-week-old mice (*n* = 5) on days 1, 3, 5, and 7. iTS-P cells showed a cobblestone-like morphology, while iF cells showed a morphology similar to that of fibroblasts (Figure 1A,B). The percentages of iPS cells, iTS-P cells, and iF cells forming colonies were 4%, 44%, and 52%, respectively (Figure 1C). iTS-P cells and iF cells grew logarithmically (Figure 1D).

The aim was to examine the teratoma formation potential and tumorigenic potential of iTS-P cells and iF cells in vivo, iTS-P, and iF cells (1 × 10^6^ cells) at passage 20 were transplanted into NOD/SCID mice. Tumors developed from iF cells but not iTS-P cells approximately 8 weeks after transplantation. Histologically, the tumors had duct-like and stromal structures but did not contain ectodermal tissue. The most prevalent histological type of tumor was pancreatic cancer instead of teratoma (Figure 1E). These data indicate that iF cells are likely to be associated with the development of pancreatic cancer.

### 3.2. Microarray Analysis

**Figure 2:**
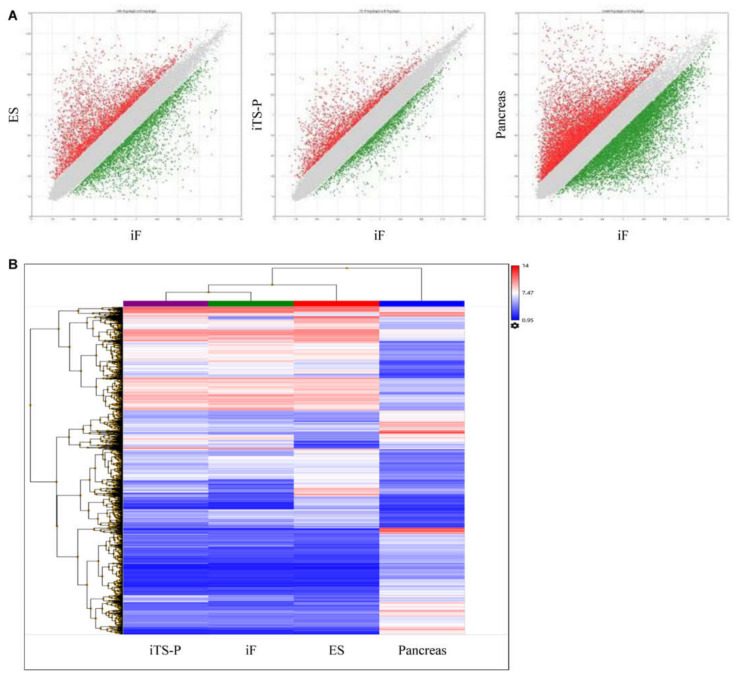
Microarray analysis. (**A**) Global gene expression patterns were compared between iF cells and ES cells, iTS-P cells, or pancreatic tissue cells (>95% islets) using a Transcriptome Analysis Console (Affymetrix). The gray area indicates genes expressed at levels differing by <2-fold between the two samples. (**B**) Unsupervised hierarchical clustering of gene expression profiles of iF cells, iTS-P cells, ES cells, and pancreatic tissue cells (>95% islets). Each column represents one biological sample.

We performed microarray analysis to compare the global gene expression profiles of ES cells, iTS-P cells (passage 30), iF cells (passage 30), and pancreatic tissue cells (>95% islets). Among 45,037 genes, the levels of 13.7% differed by >2-fold between iF cells and ES cells; the levels of 8.6% differed by >2-fold between iF cells and iTS-P cells; and the levels of 35.1% differed by >2-fold between iF cells and pancreatic tissue (Figure 2A). These data suggest that the expression pattern of iF cells was more similar to that of iTS-P cells than to that of ES cells or pancreatic tissue. Unsupervised hierarchical clustering of the gene expression profiles of ES cells, iTS-P cells, iF cells, and pancreatic tissue showed that iF cells clustered more closely with iTS-P cells than with ES cells and pancreatic tissue cells (Figure 2B), although the phenotypes of iF cells differed markedly from those of iTS-P cells.

### 3.3. Expression of ES Cell Markers and Endoderm/Pancreatic Markers in iF Cells and iTS-P Cells

**Figure 3:**
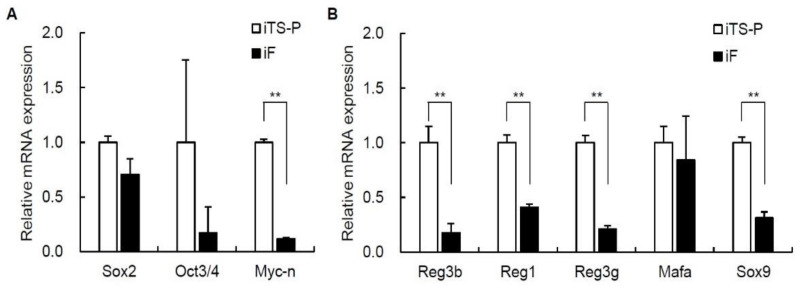
Quantitative reverse transcription-polymerase chain reaction (qRT-PCR) analysis of ES cell markers and endoderm/pancreatic markers in iF cells (passage 30) and iTS-P cells (passage 30). (**A**) iTS-P cells and iF cells were evaluated for ES cell markers using qRT-PCR. (**B**) iTS-P cells and iF cells were evaluated for endodermal/pancreatic markers using qRT-PCR. The data are expressed as the gene-to-Gapdh ratio, with this ratio for iTS-P cells arbitrarily set to 1 (*n* = 3). The error bars indicate the standard error values. ** *p* < 0.01.

To confirm the genes that determine the differences between iTS-P cells and iF cells, we performed qRT-PCR. We selected 29 ES markers, endoderm/pancreatic markers, oncogenes, intercellular adhesion markers, EMT markers, and cell growth regulatory factors from genes whose expression levels differed by more than 3-fold between iF cells and iTS-P cells in a microarray (Table 1). We first evaluated ES cell markers and endodermal/pancreatic markers in iF cells (passage 30) and iTS-P cells (passage 30). The levels of the Sox2, Oct3/4, and Myc-*n* genes differed by more than 3-fold between these cells in the microarray, and the level of only Myc-*n* was significantly higher in iTS-P cells than in iF cells (Figure 3A). The expression levels of the Reg3b, Reg1, Reg3g, Mafa, and Sox9 genes differed by more than 3-fold between these cells in the microarray; in addition, the expression levels of the Reg3b, Reg1, Reg3g, and Sox9 genes were significantly higher in iTS-P cells than in iF cells (Figure 3B).

### 3.4. Expression of Oncogenes and Intercellular Adhesion Markers in iF Cells and iTS-P Cells

**Figure 4:**
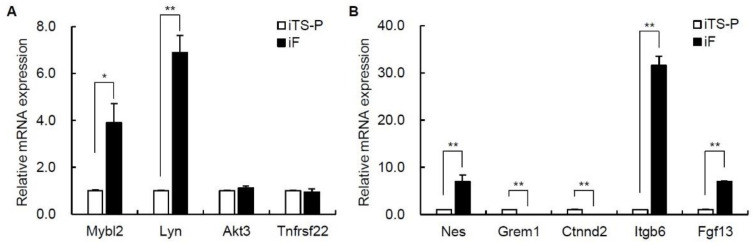
qRT-PCR analysis of oncogenes and intercellular adhesion markers in iF cells (passage 30) and iTS-P cells (passage 30). (**A**) iTS-P cells and iF cells were evaluated for oncogenes using qRT-PCR. (**B**) iTS-P cells and iF cells were evaluated for intracellular adhesion markers using qRT-PCR. The data are expressed as the gene-to-Gapdh ratio, with this ratio for iTS-P cells arbitrarily set to 1 (*n* = 3). The error bars indicate the standard error values. * *p* < 0.05, ** *p* < 0.01.

We next evaluated oncogenes and intercellular adhesion markers in iF cells (passage 30) and iTS-P cells (passage 30). Among oncogenes, the gene expression levels of Mybl2, Lyn, Akt3, and Tnfrsf22 differed by more than 3-fold between these cells in the microarray (Table 1), and the expression levels of the Mybl2 and Lyn genes were significantly higher in iF cells than in iTS-P cells (Figure 4A). Among intercellular adhesion markers, the expression levels of the Nes, Grem1, Ctnnd2, Itgb6, and Fgf13 genes differed by more than 3-fold between these cells in the microarray; in addition, the gene expression levels of Nes, Itgb6, and Fgf13 were significantly higher in iF cells than in iTS-P cells, while those of Grem1 and Ctnnd2 were significantly higher in iTS-P cells than in iF cells (Figure 4B).

### 3.5. Expression of Epithelial-Mesenchymal Transition (EMT) Markers and Cell Growth Markers in iF Cells and iTS-P Cells

**Figure 5:**
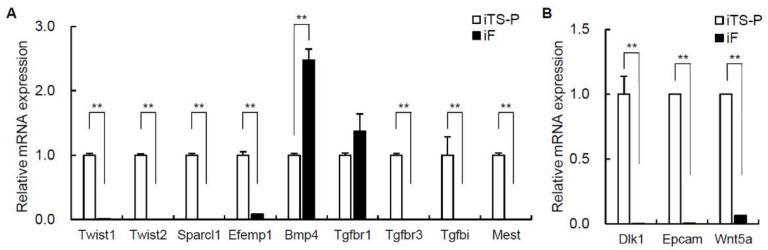
qRT-PCR analysis of epithelial-mesenchymal transition (EMT) markers and cell growth markers in iF cells (passage 30) and iTS-P cells (passage 30). (**A**) iTS-P cells and iF cells were evaluated for EMT markers using qRT-PCR. (**B**) iTS-P cells and iF cells were evaluated for cell growth markers using qRT-PCR. The data are expressed as the gene-to-Gapdh ratio, with this ratio for iTS-P cells arbitrarily set to 1 (*n* = 3). The error bars indicate the standard error values. ** *p* < 0.01.

We evaluated EMT markers and cell growth markers in iF cells (passage 30) and iTS-P cells (passage 30). Among EMT markers, the gene expression levels of Twist1, Twist2, Sparcl1, Efemp1, Bmp4, Tgfbr1, Tbfbr3, Tbfbi, and Mest differed by more than 3-fold between these cells in the microarray (Table 1); in addition, the gene expression levels of Twist1, Twist2, Sparcl1, Efemp1, Tbfbr3, Tbfbi, and Mest were significantly higher in iTS-P cells than that in iF cells, while only that of Bmp4 was significantly higher in iF cells than in iTS-P cells (Figure 5A). Among cell growth markers, the gene expression levels of Dlk1, Epcam, and Wnt5a differed by more than 3-fold between these cells in the microarray, and the expression levels of all three of these genes were significantly higher in iTS-P cells than in iF cells (Figure 5B).

### 3.6. Expression of the Mybl2, Lyn, Nestin, Itgb6, and Fgf13 Genes in iF Cells and iTS-P Cells

**Figure 6:**
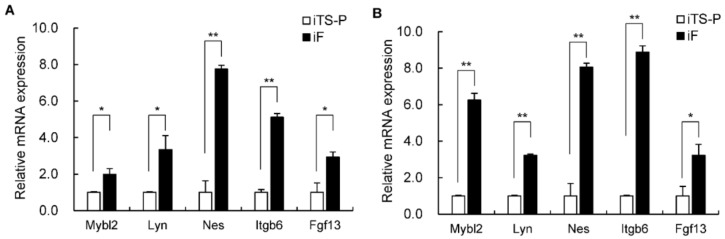
qRT-PCR analysis of the Mybl2, Lyn, Nestin, Itgb6, and Fgf13 genes in iF cells and iTS-P cells at different passages (passage 15) and from different clones (dc-). (**A**) iTS-P cells (passage 15) and iF cells (passage 15) were evaluated for the Mybl2, Lyn, Nestin, Itgb6, and Fgf13 genes using qRT-PCR. (**B**) dc- iTS-P cells and dc-iF cells were evaluated for the Mybl2, Lyn, Nestin, Itgb6, and Fgf13 genes using qRT-PCR. The data are expressed as the gene-to-Gapdh ratio, with this ratio for iTS-P cells arbitrarily set to 1 (*n* = 3). The error bars indicate the standard error values. * *p* < 0.05, ** *p* < 0.01.

We evaluated the expression of the Mybl2, Lyn, Nestin, Itgb6, and Fgf13 genes, which could be important biomarkers to distinguish iTS-P cells from iF cells, in iF cells and iTS-P cells at different passages (passage 15) and from different clones (dc-). The gene expression levels of these genes were significantly higher in the iTS-P cells (passage 15) than in the iF cells (passage 15) (Figure 6A). The gene expression levels of these genes were significantly higher in the dc- iTS-P cells than in the dc-iF cells (Figure 6B).

## 4. Discussion

We previously reported the generation of iTS-P cells and iF cells, which are capable of self-renewal, via transient overexpression of reprogramming factors [18,20,32]. iTS-P cells are similar to pancreatic stem cells, while iF cells behave similarly to pancreatic cancer cells. In this study, we analyzed the expression of a total of 45,037 genes in iTS-P cells and iF cells by microarray analysis. The gene expression profile of iF cells was more closely related to that of iTS-P cells than to that of ES cells or pancreatic tissue cells. Although the morphologies of iF cells and iTS-P cells are markedly different, the gene expression levels were largely similar, suggesting that a few genes may determine the differences between iTS-P cells and iF cells.

The most likely factors that determine the differences between iTS-P cells and iF cells may be oncogenes. The expression levels of the Mybl2 and Lyn genes were significantly higher in iF cells than in iTS-P cells. Mybl2 is a member of the Myb family of transcription factors and an important physiological regulator of cell cycle progression, cell survival, and cell differentiation. On the other hand, deregulation of Mybl2 expression is involved in cancer initiation and progression, and high Mybl2 expression is significantly correlated with poor patient outcome in numerous cancers [37]. Lyn is a member of the Src tyrosine kinase family and functions as a proto-oncogene in tumor progression. Lyn is frequently overexpressed in numerous tumor types, including chronic myelogenous leukemia, renal cancer, cervical cancer, head and neck squamous cell carcinoma, gastric cancer, and prostate cancer [38]. High expression of Mybl2 and Lyn may be associated with the characteristics of iF cells. On the other hand, the expression levels of four major driver genes for pancreatic cancer (KRAS, CDKN2A, TP53, and SMAD4) [39] differed by less than 3-fold between the iF cells and the iTS-P cells in a microarray. It has been reported that inactivating mutations in tumor suppressor genes such as CDKN2A/p16, TP53, and SMAD4 cooperate with KRAS mutations to cause aggressive tumor growth of pancreatic cancer [40]. These four genes are unlikely to participate in the generation of iF cells, because iTS-P cells and iF cells theoretically have the same genes.

Nestin is a class VI intermediate filament protein originally found in neuroepithelial stem cells and neural cells. It has been reported that Nestin is expressed in not only pancreatic progenitor cells but also pancreatic ductal adenocarcinomas [41,42]. Itgb6 is a membrane-spanning heterodimeric glycoprotein involved in wound healing and the pathogenesis of diseases, including fibrosis and cancer [43]. The possible involvement of Fgf13 in cancer has been reported. Overexpression of Fgf13 is associated with shorter progression-free survival times in pancreatic cancer [44] and poor outcome in cervical cancer [45]. High expression of Nestin, Itgb6, and Fgf13 may also be associated with the characteristics of iF cells.

We first suspected that iF cells might be pancreatic cancer cells in which EMT was induced [46,47,48,49,50], because the morphology of iF cells was fibroblast-like. However, the expression of only one EMT marker (Bmp4) was significantly higher in iF cells than in iTS-P cells, while the expression of Twist1, Twist2, Sparcl1, Efemp1, Tbfbr3, Tbfbi, and Mest was significantly higher in iTS-P cells than in iF cells. Therefore, iF cells are unlikely to be cells in which EMT was induced. Indeed, the expression of Epcam, which is a type I transmembrane glycoprotein, in iTS-P cells was significantly higher than that in iF cells, and it has been reported that loss of Epcam expression coincides with a gain in vimentin expression (mesenchymal marker) in all tumor cells, consistent with the first switch to the mesenchymal state [35]. High expression of Epcam in iTS-P cells may inhibit EMT induction.

Figure 5B shows significantly higher expression of the proliferative markers Dlk1 and Wnt5a in iTS than in iF cells and an apparently lower proliferative capacity in vitro of iTS cells than iF cells in Figure 1D. Dlk1 is mainly known for its involvement in adipogenesis, although it has been associated with many other stem cells/progenitors and is known to be widely expressed during organism development and tissue regeneration [51]. Dlk1 is expressed in a subpopulation of hepatic oval cells, which are considered stem/progenitor cells in rat adult liver [52]. Wnt proteins are a group of secreted signaling proteins that function to regulate cell fate and pattern formation during embryogenesis. The Wnt5a signaling pathway plays a key role in proper insulin cell migration and the development of pancreatic islets [53]. Altered expression of Wnt5a has been implicated in human carcinogenesis and tumor progression, including that of pancreatic cancer [54]. The reason for the evident contradiction in Figure 1D and Figure 5B is unknown. Further investigations of the relationship between iTS-P/iF cells and Dlk1/Wnt5a are needed.

## 5. Conclusions

The Mybl2, Lyn, Nestin, Itgb6, and Fgf13 genes could be important biomarkers to distinguish iTS-P cells from iF cells. Although the generation of iPS/iTS cells has important implications due to their potential use in cell replacement therapy, this approach is also associated with the risk of generating cancer-like cells through reprogramming. The selection of iPS/iTS cells from other cells, including iF cells, is one of the important steps in applying these cells in clinical settings.

## Figures and Tables

**Table 1:** Twenty-nine ES marker, endoderm/pancreatic marker, oncogenic, intercellular adhesion marker, EMT marker, and cell growth regulatory factor genes whose expression levels differed by >3-fold between iF cells and iTS-P cells.

	iTS-P Ave (log2)	iF Ave (log2)	Fold Change
**ES marker**			
Sox2	5.39	3.67	3.3
Oct3/4	6.98	4.76	4.66
Myc-*n*	5.68	2.92	6.8
**Endoderm/Pancreatic marker**			
Reg3b	12.56	4.16	337.13
Reg1	11.69	4.52	143.91
Reg3g	10.1	4.41	51.61
Mafa	5.39	3.67	3.3
Sox9	7.11	5.26	3.63
**Oncogene**			
Mybl2	5.18	7.21	−4.09
Lyn	5.28	7.29	−4.03
Akt3	3.88	5.73	−3.62
Tnfrsf22	4.58	6.82	−4.75
**Intercellular adhesion marker**			
Nes	2.08	6.49	−21.27
Grem1	9.72	4.55	35.85
Ctnnd2	4.64	2.5	4.42
Itgb6	3.65	6.76	−8.61
Fgf13	3.42	6.42	−7.98
**EMT marker**			
Twist1	6.48	3.77	6.53
Twist2	6.18	4.2	3.93
Sparcl1	4.39	2.38	4.03
Efemp1	10.76	6.65	17.32
Bmp4	5.47	7.09	−3.08
Tgfbr1	3.99	7.34	−10.15
Tgfbr3	8.53	4.22	19.82
Tgfbi	4.97	2.06	7.51
Mest	6.66	4.23	5.41
**Cell growth regulation factor**			
Dlk1	8.83	4.26	23.66
Epcam	10.63	4.14	90.04
Wnt5a	5.87	2.3	11.88

## Data Availability

The data that support the findings of this study are available from the corresponding author, H.N., upon reasonable request.
